# Rust

**Published:** 1857-08

**Authors:** 


### RUST

Is best removed from knives, &c., by rotten-stone and oil; it is prevented as to stoves and grates, when put away in damp places for the summer time, by painting them with a mixture of three parts of lard and one part of rosin, melted together; but the best way to keep men from rusting, is to give them steady employment of an active, pleasurable, and profitable character; without this, the health declines, the mind is enervated, and life itself is eaten out before its time.

Better far, to wear out in moderate and useful activities, than to rust out in inglorious ease. Sun, moon and stars; air, earth and ocean; rill and river; cascade and cataract; all, by their ceaseless motion, live. There is not an atom wholly idle in the wide universe. Nor should man be. They are for time. He for eternity. Their destiny is fixed for them. Man makes his own, according to the work of his hands.

